# The Effect of Swiss Chard Powder as a Curing Agent on Volatile Compound Profile and Other Qualitative Properties of Heat-Treated Sucuk

**DOI:** 10.3390/foods14213785

**Published:** 2025-11-04

**Authors:** Betül Katmer, Mükerrem Kaya

**Affiliations:** Department of Food Engineering, Faculty of Agriculture, Atatürk University, Erzurum 25240, Türkiye; mkaya@atauni.edu.tr

**Keywords:** heat-treated sucuk, Swiss chard powder, natural curing agent, volatiles, fermented sausage, lipid oxidation, sensory

## Abstract

The aim of the study was to determine the effect of Swiss chard powder (SCP) as a natural nitrite source on the volatile compounds and other qualitative properties of heat-treated sucuk (HTS). Three formulations were created for the production of HTS: control (no nitrite addition), synthetic nitrite (SN, 150 mg/kg NaNO_2_ addition), and natural nitrite from Swiss chard powder (SCPN) (SCP equivalent to 150 mg/kg NaNO_2_). The HTS production was carried out under controlled conditions. Physicochemical and microbiological properties of the HTS were investigated during the production stages. The final product was analyzed for volatile compounds, residual nitrite, and sensory properties. A higher mean pH value was found in the SCPN group in comparison with other groups (*p* < 0.05). In all production stages, the lowest a_w_ values were observed in the presence of SCPN (*p* < 0.05). The highest mean L* value was determined in the group with SN (*p* < 0.05). Groups containing SN or SCPN exhibited higher a* values compared to the control during fermentation, heat treatment, and drying. The SN group had the lowest TBARS value during all these stages (*p* < 0.05). There was no significant difference in the amount of residual nitrite between the SCPN and SN groups (*p* > 0.05). In terms of sensory parameters, nitrite groups (SCPN and SN) had higher values than the control group (*p* < 0.05). Lactic acid bacteria exhibited good growth during fermentation in all groups. Although SCP positively affected many volatile compounds, this effect was not strong enough to alter the sensory properties of the product. Correlation analysis of volatile compounds revealed that the control group was significantly different from the groups using SN or SCPN. Additionally, similar characteristics in volatile compounds and sensory attributes were observed in the SN and SCPN groups. As a result, characteristics of the final products were not usually adversely affected by the use of SCP in HTS production.

## 1. Introduction

Fermented sausages are produced as dry and semi-dry fermented sausages and the quality of these products depends on many variables related to the raw materials used, microorganism population and processing conditions [1]. Nitrate is used for the production of fermented sausages with long ripening times, whereas nitrite is usually preferred for fermented sausages with short ripening times [2]. While nitrite is the primary curing agent, nitrate requires conversion to nitrite to exert the desired effects in the product. Nitrite is effective in inhibiting foodborne pathogens and improving color formation and stability. This curing agent additionally contributes to delaying oxidation and determining the cured flavor [2,3]. Furthermore, nitrite also plays a role in the formation of nitrosamines, which are known carcinogens. This is a major source of concern for consumers. Thus, there is a shift towards natural products. Consequently, interest in using natural sources as an alternative to synthetic nitrates and nitrites has been increasing in the meat industry [3]. Some vegetables, such as celery, spinach, and chard, contain high levels of nitrate. Preparations made from such vegetables can be used directly as sources of nitrate. Furthermore, nitrate in these preparations is converted to nitrite by nitrate-reducing bacteria, and these products are called pre-converted plant extracts. In the meat industry, pre-converted celery powder is commonly used as a natural source of nitrite [3,4].

There are a number of studies in the literature on the use of plant extracts (powder and juice form) such as chard, celery, parsley, radish, leek powder and red beet as curing agents in different types of fermented sausages such as salami, chorizo, and Italian-type dry sausage, as well as on the effects of plant extracts on the physicochemical properties, microbiological and sensory attributes of products have been investigated [5,6,7,8,9,10,11,12,13]. Studies have also investigated the application of plant-based curing alternatives in two varieties of Turkish fermented sausage: sucuk and heat-treated sucuk (HTS). The effects of beet root, dill, spinach, celery and Swiss chard on product properties of sucuk (a dry-fermented sausage) are also assessed [14,15,16].

The production of HTS, a semi-dry-fermented sausage, involves fermentation followed by heat treatment and a short drying period. Due to its short production time, the use of nitrite is allowed in the manufacture of this product, and according to the Turkish Food Codex Regulation on Food Additives, the use of nitrite (150 mg/kg) is permitted in this product [17]. The effects of pre-converted celery powder investigated as a nitrite source on the product’s physicochemical properties, as well as on nitrosamines and volatile compounds was investigated [18]. In a study using chard powder in the manufacture of HTS, it was reported that additional ingredients or innovative applications were needed to use chard as a nitrate source in HTS production, based on the results of the physicochemical analysis [19]. In another study, the effect of using pre-converted Swiss chard powder (SCP) as an alternative curing agent in HTS production on nitrosamine formation and some quality parameters of the product was determined [20]. However, in these studies on the usability of Swiss chard in HTS, the effects of this natural product during the production process were not investigated. In addition, more research into the influence of SCP on the volatile compounds of HTS and other fermented sausages is needed. When using alternative plant-based curing agents in meat products, sensory characteristics must be considered in addition to product safety. Sensory parameters such as flavor, aroma and overall palatability may significantly affect consumer satisfaction [3,21]. Plant-based curing agents in fermented sausages can also affect the aroma through complex interactions during fermentation [21]. Therefore, it is thought that the profile of volatile compounds should also be determined in fermented sausages produced by using plant-based curing agents. This study aimed to investigate the effects of pre-converted SCP as a curing agent (nitrite) on the volatile compound profile of HTS. Another aim was to highlight the effects of SCP on other quality characteristics of HTS during production stages.

## 2. Materials and Methods

### 2.1. Material

In the production of heat-treated sucuk (HTS), beef and beef fat were used as raw materials. The cumin, red pepper, black pepper, allspice, salt, and garlic were purchased from a local market; sodium nitrite was obtained from a commercial supplier; Swiss chard powder (SCP) (Vegstable^®^ 531) (nitrite content 14.700 mg/kg) was supplied by a commercial company (Florida-Food Products, Lake Mary, FL, USA); and collagen casings (Naturin GmbH & Co., Weinheim, Germany) were used for stuffing. In the HTS formulations, autochthonous strains (*Latilactobacillus sakei* S15 and *Staphylococcus xylosus* GM92) were used as starter culture [22,23].

### 2.2. HTS Production

The formulations were prepared according to the 80:20 (lean meat: beef fat) ratio, based on the recipe reported by Armutçu et al. [1]. For each kg of the meat–fat mixture, 20 g salt, 9 g cumin, 10 g garlic, 7 g red pepper, 5 g black pepper, 2.5 g allspice, and 4 g sucrose were used. In each production trial, three different batters were prepared. In the first group (control), neither synthetic nitrite (SN) nor Swiss chard powder-derived nitrite (SCPN) was added. The second group contained 150 mg/kg sodium nitrite (SN), while the third group included chard powder as a nitrite source equivalent to 150 mg/kg sodium nitrite (SCPN). The study was repeated with three different batches of raw materials, resulting in a total of nine HTS batters.

The batters were prepared using a laboratory-scale bowl cutter (MTK 662, Mado, Dorhan, Schwarzwald, Germany). They were then stuffed into collagen casings with a piston-type stuffer (Mado MTK-591, Dorhan, Schwarzwald, Germany) and subjected to fermentation in a climate chamber (Reich, Thermoprozesstechnik GmbH, Schechingen, Germany) at 24 °C for 24 h with 92 ± 2% relative humidity. Following this step, the heat treatment was applied to samples in a cooking chamber (Mauting, Valtice, Czech Republic) until they reached a core temperature of 64 °C. After this treatment, drying was applied to the samples in a climate chamber (16 °C, 86–88% relative humidity, 4 days).

### 2.3. Microbiological Analyses

Lactic acid bacteria, Enterobacteriaceae, and *Micrococcus*/*Staphylococcus* counts were determined on de Man Rogosa Sharpe Agar (Merck, Darmstadt, Germany), Violet Red Bile Dextrose Agar (Merck), and Mannitol Salt Phenol Red Agar (Merck), respectively. The plates for lactic acid bacteria and Enterobacteriaceae were incubated under anaerobic conditions at 30 °C for 48 h, and *Micrococcus*/*Staphylococcus* plates were incubated at 30 °C for 48 h. Yeasts and molds were enumerated on Rose Bengal Chloramphenicol Agar (Merck), followed by incubation at 25 °C for 5 days [24].

### 2.4. Physicochemical Analyses

#### 2.4.1. pH and Water Activity (a_w_)

For pH measurement, 10 g of each sample and final product was homogenized with 100 mL distilled water using an ultra turrax homogenizer (IKA Werke T25, Staufen im Breisgau, Germany) for 1 min, and the pH was measured with a pH meter (Mettler Toledo, Switzerland) [18]. Water activity was determined using an a_w_ meter (Novasina, TH/500, Pfäffikon, Switzerland).

#### 2.4.2. Thiobarbituric Acid Reactive Substances (TBARS)

TBARS values were determined according to the method described by Kılıç and Richards [25]. Two grams of each sample was mixed with TCA solution and homogenized for 15–30 s. The mixture was filtered through Whatman No. 1 filter paper, and 3 mL of filtrate was transferred to a test tube, followed by the addition of 3 mL of 0.02 M thiobarbituric acid solution. After heating in a boiling water bath for 40 min, the samples were cooled for 5 min and centrifuged at 2000 rpm for 5 min. Absorbance was measured at 532 nm, and the results were expressed as mg MDA/kg.

#### 2.4.3. Residual Nitrite

Residual nitrite content was determined according to the NMKL method [26]. Ten grams of homogenised sample was mixed with 50 mL of ultrapure water (50–60 °C) and subsequently with 50 mL of acetonitrile, followed by gentle mixing. The mixture was cooled to room temperature and diluted to a final volume of 200 mL with ultrapure water. The solution was first filtered through nitrite/nitrate-free filter paper, and then through a 0.45 μm disposable membrane filter for final clarification. The analysis was performed using HPLC/DAD (Agilent Technologies, Santa Clara, CA, USA). Separation was achieved on a Hamilton PRP-X100 column (5 μm, 150 × 4.6 mm, Hamilton company, Reno, NY, USA) with a flow rate of 2 mL/min. The injection volume was 100 μL, and detection was carried out at 220 nm. Results were expressed as mg/kg.

For method validation, standard nitrite solutions (5–30 mg/L) were spiked into the samples at five concentration levels, each analyzed in quintuplicate. The mean recoveries of nitrite ranged between 99.20% and 103.04%, with relative standard deviations (RSDs) from 1.60% to 2.83%. The limits of detection (LOD) and quantification (LOQ) were determined using standard solutions at concentrations of 1, 3, 5, 10, and 20 mg/L, based on the equations LOD = 3.3 × Sy/s and LOQ = 10 × Sy/s, where *Sy* represents the standard deviation of the response and *s* denotes the slope of the calibration curve. The calibration curve for nitrite showed a good linearity with a correlation coefficient (R^2^) of 0.9999. The LOD and LOQ values for nitrite were 1.01 mg/L and 3.06 mg/L, respectively.

#### 2.4.4. Instrumental Color

The color parameters (L*: lightness, a*: red-green and b*: yellow-blue) of the HTS cross-sections were measured using a colorimeter (CR-400, Minolta Co., Osaka, Japan) with an illuminant D65, 8 mm aperture size, and 2° observation angle.

### 2.5. Sensory Analysis

Sensory analysis was carried out using a semi-trained panel comprising 25 members. The panelists were informed about sampling, analyzing and interpreting stimuli, and the corresponding scores. They were also given detailed instructions on the evaluation form and the evaluation procedures. The training consisted of an explanatory session in which the objectives of the tests were presented to the panelists. A monadic test was applied to the samples and the panelists were asked to evaluate the samples separately. A 9-point hedonic scale (1: very bad, 9: very good) was used to evaluate five attributes: color, odor, taste, texture, and overall acceptability. The samples were sliced into about 1 cm thick portions and randomly presented at room temperature (22 ± 2 °C) under standardized lighting conditions. Panelists were instructed to cleanse their palate with water and unsalted bread between samples, maintaining a minimum interval of 30 s to prevent sensory fatigue and carryover effects. The samples were evaluated in three different sessions and on three different days, and a total of 75 sensory analysis results were obtained for each parameter.

### 2.6. Volatile Compounds

Volatile compounds were extracted by weighing 5 g of sample into a 40 mL vial, which was held in a thermal block at 30 °C for 1 h to allow equilibrium between the sample matrix and the headspace. A 75 μm Carboxen/Polydimethylsiloxane (CAR/PDMS) fiber (Supelco, Bellefonte, PA, USA) was then exposed to the headspace for 2 h to adsorb compounds. Following extraction, the fiber was inserted into the port of a gas chromatograph/mass spectrometer (GC 6890N/MS 5973, Agilent, Santa Clara, CA, USA) and desorbed for 6 min at 250 °C in splitless mode. Separation was achieved on a DB-624 capillary column (30 m × 0.25 mm × 1.4 μm, J&W Scientific, Folsom, CA, USA) using helium as the carrier gas at a constant flow rate of 1 mL/min. The GC oven was programmed as follows: 40 °C (5 min), ramped to 110 °C at 3 °C/min, then to 150 °C at 4 °C/min, and finally to 210 °C at 10 °C/min, where it was held for 12 min. The GC/MS interface was held at 280 °C. The mass spectra were obtained in electron impact mode at 70 eV, with an acquisition range of 30 to 400 amu. The volatile compounds were identified by comparing the mass spectral data obtained from the Wiley, NIST and Flavor libraries with the retention time and mass spectra of the authentic compounds. Kovats’ indexes were determined using a paraffin mix (Supelco 44585-U, Bellefonte, PA, USA) and compared with those reported in the literature. The results were reported in arbitrary units (AU) × 10^6^ [27].

### 2.7. Statistical Analysis

The study design included three treatment groups (C: without synthetic or chard powder-derived nitrite; SN: 150 mg/kg NaNO_2_; SCPN: Swiss chard powder equivalent to 150 mg/kg NaNO_2_). Experiments were performed in a factorial design with a randomized complete block design and three replications (three batters for each treatment). All measurement were repeated at least two times using duplicate samples. Data were analyzed using two-way ANOVA, and means showing statistically significant variation sources and interactions were compared using Duncan’s multiple range test. The results are given as mean ± standard deviation in tables and figures. To determine relationship between treatments and volatile compounds, the correlation heatmap was carried out using ChiPlot [28]. To determine the relationship between treatment, volatile compounds and sensory properties, PCA was applied using the Minitab 17.1.0 software (State College, PA, USA).

## 3. Results and Discussion

### 3.1. The Effect of Using SCP on pH

**Figure 1:**
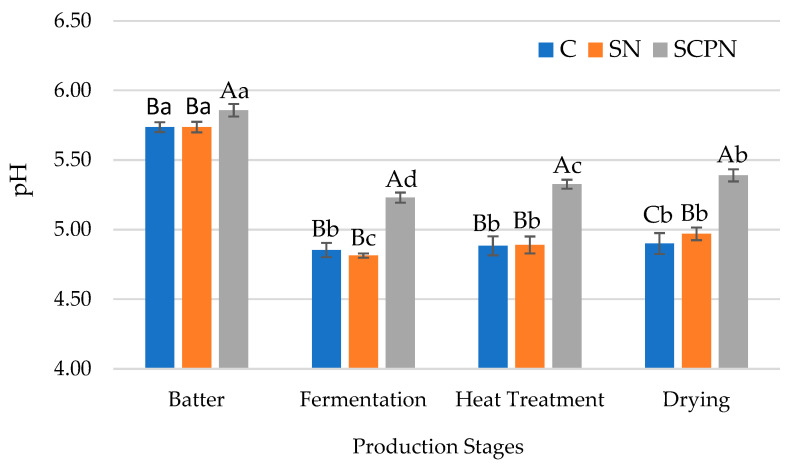
The effect of treatment × production stage interaction on pH value of HTS (C: Control, SN: Synthetic nitrite, SCPN: Swiss chard powder nitrite) (mean ± standard deviation) (*n* = 36) (*p* < 0.05). a–d: Different lowercase letters indicate significant differences between product stages for each treatment. A–C: Different uppercase letters indicate significant differences between treatments for each production stage.

The use of treatment and the production stage had a very significant effect (*p* < 0.01) on the pH of the HTS (Table 1). The interaction between treatment and production stage was also found to be statistically highly significant (*p* < 0.01). As can be seen in Figure 1, at the initial stage (HTS batter), Swiss chard powder-derived nitrite (SNCP) group had higher average pH value than the control (C) and synthetic nitrite (SN) groups. This result can be attributed to the high pH value of SCP, which consequently increased the initial pH of HTS. Similar results were observed at other stages of production (fermentation, heat treatment and drying) (Figure 1). The pH value decreased in all groups during the fermentation phase due to the use of a starter culture. However, the pH value increased during the heat treatment phase (Figure 1). Ercoşkun et al. [29] reported that this increase was due to protein denaturation. It is also stated that the increase in pH with heat treatment is related to the reduction of carboxyl group amount in proteins and the release of calcium and magnesium ions from proteins [30]. A slight pH increase was also observed during drying stage. However, this increase in pH was found to be statistically significant only in the presence of SCPN (Figure 1). The increase in pH value during the drying stage may be due to accumulation of alkaline substances by proteolysis [31].

### 3.2. The Effect of Using SCP on a_w_

**Figure 2:**
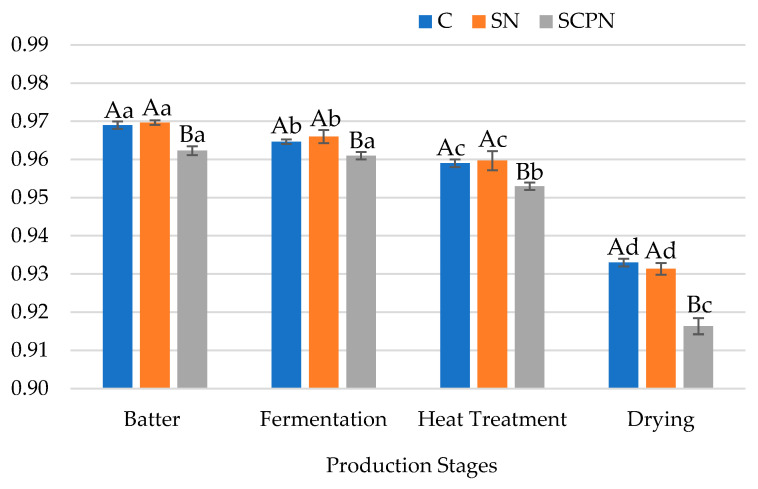
The effect of treatment × production stage interaction on a_w_ value of HTS (C: Control, SN: Synthetic nitrite, SCPN: Swiss chard powder nitrite) (mean ± standard deviation) (*n* = 36) (*p* < 0.05). a–d: Different lowercase letters indicate significant differences between product stages for each treatment. A–B: Different uppercase letters indicate significant differences between treatments for each production stage.

Significant effects (*p* < 0.01) of treatment and production stage on the a_w_ value of the HTS were observed (Table 1). In addition, the interaction of these two factors also had an effect on the a_w_ value. As seen in Figure 2, both in the HTS batter and during the other production stages, HTS samples produced with SCPN showed lower a_w_ values compared to the other groups. However, in the drying stage, the difference between the SCPN group and the other groups was greater compared to the other stages. In a study conducted on the use of different levels of SCP in the production of HTS, it was also reported that samples cured with SCP were reported to have lower a_w_ values than the control [20]. Similarly, in a study investigating the potential of celery powder as a curing agent in HTS, it was reported that increasing the proportion of celery powder resulted in lower a_w_ values in the final product [18]. In another study on sucuk, it has also been reported that sucuk samples prepared with celery powder exhibited lower a_w_ values than the control group [16]. The decrease in a_w_ values during the ripening period of fermented sausages, together with the reduction in pH, plays an important role in producing safer products with longer shelf life [32].

### 3.3. The Effect of Using SCP on TBARS

**Figure 3:**
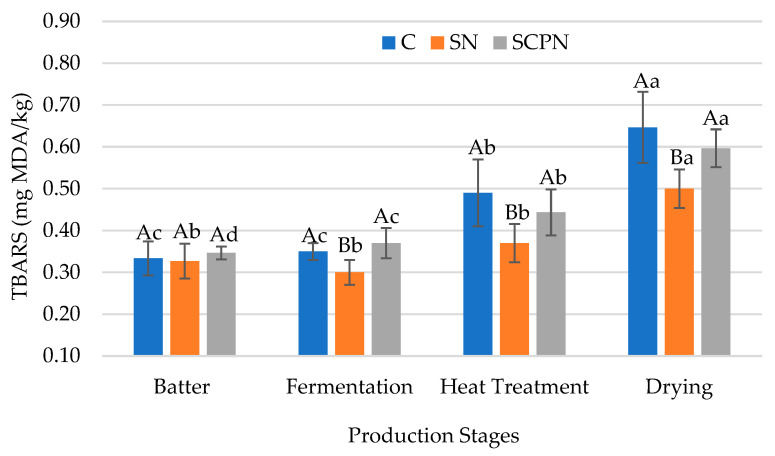
The effect of treatment × production stage interaction on TBARS value of HTS (C: Control, SN: Synthetic nitrite, SCPN: Swiss chard powder nitrite) (mean ± standard deviation) (*n* = 36) (*p* < 0.05). a–d: Different lowercase letters indicate significant differences between product stages for each treatment. A–B: Different uppercase letters indicate significant differences between treatments for each production stage.

The lowest mean TBARS value was observed in the treatment group with SN (Table 1). In addition, the interaction of two factors also had an effect on the TBARS value (*p* < 0.05). As shown in Figure 3, no significant difference was observed among the groups in terms of TBARS value in the HTS batter. At the other production stages, the lowest TBARS value was observed in HTS samples cured with SN; however, there was no statistical difference between the control and SCPN groups in terms of TBARS value in the production (Figure 3). These results indicate that SN is an important additive in preventing lipid oxidation [3].

### 3.4. The Effect of Using SCP on Residual Nitrite

Residual nitrite is the amount of nitrite present in the meat matrix that does not react with myoglobin, the color pigment of meat [33]. In this study, the SCPN group showed the highest residual nitrite content; however, the difference between the mean values of the SN and SCPN groups was statistically insignificant (*p* > 0.05) (Table 1). Yılmaz Oral [20] also reported comparable results. In the present study, a lower residual nitrite amount was detected in the control group. During manufacture, nitrate derived from spices can be converted into nitrite [34], and it is considered that this low residual nitrite content originated from spices [35].

### 3.5. The Effect of Using SCP on Instrumental Color

A significant effect of the nitrite source on L* value was detected (*p* < 0.05), and the SN group exhibited the highest L* value (Table 1). SCPN showed the lower L* value than SN group. Similarly, research conducted by Ozaki et al. [12], Horsch et al. [36], Xi et al. [37] and Djeri and Williams [38] also reported that products used as nitrite alternatives caused a decrease in the L* value. While a reduction in L* value was observed at the fermentation stage, a significant increase was recorded during heat treatment stage (Table 1). The decrease in L* value during fermentation has been attributed to the formation of dark color resulting from browning reactions occurring at this stage [39,40,41]. Similar observations was also reported by Ercoşkun et al. [29].

**Figure 4:**
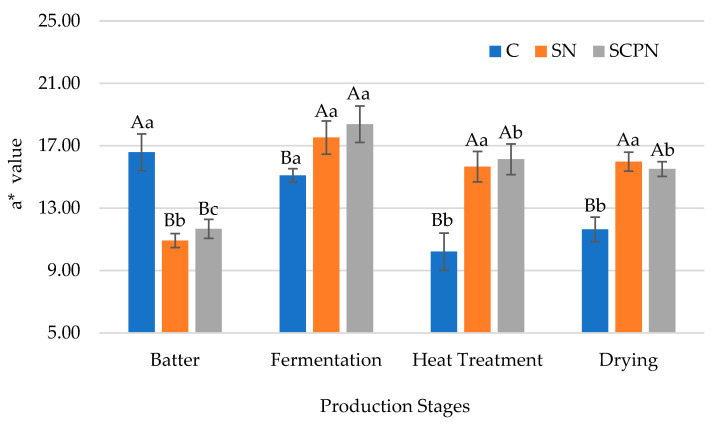
The effect of treatment× production stage interaction on a* value of HTS (C: Control, SN: Synthetic nitrite, SCPN: Swiss chard powder nitrite) (mean ± standard deviation) (*n* = 36) (*p* < 0.05). a–c: Different lowercase letters indicate significant differences between product stages for each treatment. A–B: Different uppercase letters indicate significant differences between treatments for each production stage.

The a* value of HTS was strongly influenced by nitrite source and production stage. Furthermore, their interaction also had a very significant effect on the a* value (*p <* 0.01) (Table 1). At the initial stage (batter), the control showed higher a* value than the other groups. In contrast, at the other stages, higher a* values were observed in the SN and SCPN groups (Figure 4). This result is considered to be due to the formation of nitrosomyoglobin, which occurs as a result of the reaction between nitrite and myoglobin during the fermentation stage [34]. During the fermentation stage, the a* value increased in the SN and SCPN groups. No significant change was observed in the a* value during the heat treatment and drying stage in the SN group, while a* value decreased in the control and SCPN groups during these stages (Figure 4). This results clearly indicated the effect of synthetic nitrite on the color of the cured meat products. On the other hand, the heat treatment applied in HTS forms stable nitroso-hemochrome [34,42].

**Figure 5:**
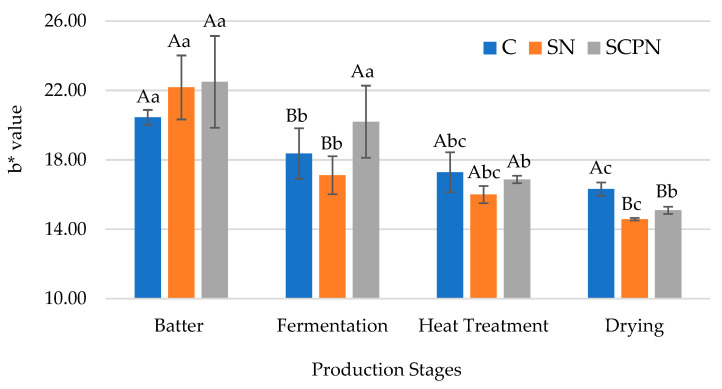
The effect of treatment × production stage interaction on b* value of HTS (C: Control, SN: Synthetic nitrite, SCPN: Swiss chard powder nitrite) (mean ± standard deviation) (*n* = 36) (*p* < 0.05). a–c: Different lowercase letters indicate significant differences between product stages for each treatment. A–B: Different uppercase letters indicate significant differences between treatments for each production stage.

SCP application had no significant impact on the b* value (*p* > 0.05), whereas the nitrite source × production stage interaction significantly influenced the b* value of HTS (*p* < 0.05) (Table 1). At the initial stage (HTS batter), no significant difference was observed between treatments in terms of b* value. At the fermentation stage, the b* value decreased in the control and SN groups. However, no change was observed in the SCPN group at this stage (Figure 5). Pérez-Alvarez et al. [43] stated that the decrease in the b* value observed during the fermentation stage of fermented sausages was due to the reduction in oxymyoglobin content, resulting from oxygen consumption during the exponential growth phase of microorganisms. As shown in Figure 5, the SCPN group exhibited a higher mean value at fermentation stage. At the end of drying, however, the control group showed a higher mean value compared to the other two groups. The mean value was higher in the SCPN group compared to the SN group; however, this difference was insignificant. Similarly, in a study conducted on Mortadella-type sausages, it was reported that parsley extract gave higher b* values compared to synthetic nitrite [44]. A study on meatballs also found that the b* value increased with rising levels of chard powder [45].

### 3.6. The Effect of Using SCP on Microbiological Properties

The influence of SCP on the microbiological characteristics of HTS is presented in Table 2. It was found that SCP, when used as a nitrite alternative, did not significantly affect the lactic acid bacteria (LAB) number (*p* > 0.05). However, the production stage factor was found to have a very significant effect (*p* < 0.01) on the LAB count (Table 2).

**Figure 6:**
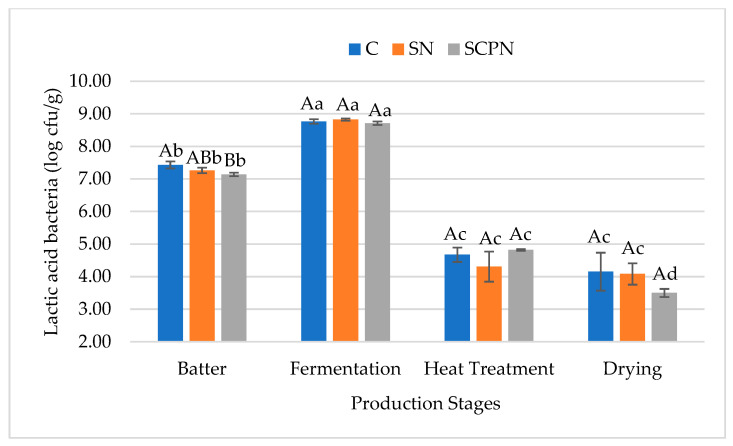
The effect of treatment × production stage interaction on lactic acid bacteria count of HTS (C: Control, SN: Synthetic nitrite, SCPN: Swiss chard powder nitrite) (mean ± standard deviation) (*n* = 36) (*p* < 0.05). a–c: Different lowercase letters indicate significant differences between product stages for each treatments. A–B: Different uppercase letters indicate significant differences between treatments for each production stages.

As shown in Figure 6, at the initial stage, the SCPN group exhibited a lower LAB count compared to the control group, whereas no significant differences were observed among the treatment groups at the other subsequent stages. There was no significant difference in the number of LAB during the heat treatment and drying stages in the control and SN groups. However, in the SCPN group, the number decreased during the drying stage (Figure 6). Similarly, Yılmaz Oral et al. [18] reported lower LAB count in HTS samples produced with celery powder, equivalent to 150 mg/kg sodium nitrite compared to the control group. These differences in LAB count are thought to be due to factors such as pH and a_w_. In the present study, LAB grow adequately in all groups, leading to a decrease in pH. In fermented sausages such as HTS, the reduction in pH during the fermentation stage is of great importance both for product safety and for the development of sensory characteristics [1,46,47].

The use of SCPN decreased the *Micrococcus*/*Staphylococcus* count of HTS (*p* < 0.05) (Table 2). A similar result was also observed in sausage samples produced with celery powder [47]. This group of microorganisms showed very slow growth during the fermentation stage of HTS. Previous studies have also demonstrated that these acid-sensitive microorganisms either grow very little or not at all during the fermentation stage of fermented sausages [1,48,49]. A reduction was observed during the heat treatment and drying stages (Table 2). According to the results obtained, the counts of Enterobacteriaceae and yeast-mold in all groups during the production stages were found to be below the limit level (<2 log cfu/g). Comparable results have also been reported in other studies on HTS [1,48]. In HTS, low pH and water activity are important inhibitory factors in controlling *Enterobacteriaceae* group microorganisms [50,51].

### 3.7. The Effect of Using SCP on Sensory Attributes

According to the results, significant differences (*p* < 0.05) were found between the control group and the SN and SCPN groups in terms of sensory attributes; however, no statistical differences (*p* > 0.05) were observed between the samples containing SN and those containing SCP. The control group showed the lowest sensory scores (Table 3). These findings indicate that nitrite is an indispensable additive for the development of desirable sensory attributes, particularly color and flavor, in meat products [2,3,34]. In the present study, the absence of differences in sensory characteristics between the SN and SCPN groups indicates that this natural product has the potential to be used as an alternative curing agent in HTS. Furthermore, a study conducted with SCP reported that its addition enhanced the taste and overall acceptability parameters of HTS [20]. On the other hand, research on sausages demonstrated that the use of paprika powder, blueberry, and celery powder positively influenced the sensory properties of the products, indicating that these natural ingredients may be suitable as curing agents in sausage production [52].

### 3.8. The Effect of Using SCP on Volatile Compounds

In the HTS groups produced in this study, a total of 52 different volatile compounds belonging to eight different chemical groups were identified including 5 alcohols, 4 aldehydes, 1 ketone, 1 acid, 10 sulfur-containing compounds, 26 terpenes, 1 furan, and 4 aromatic hydrocarbons (Table 4). The lowest ethanol abundance was observed in the presence of SN. The use of SCP also led to an increase in the levels of 1-propen-2-ol and 2-propen-1-ol (Table 3). This result indicates that these compounds may formed only in the presence of SN and SCPN. Furthermore, this finding points to the importance of nitrite in the development of cured flavor in fermented products [34].

In the HTS groups, four different aldehydes were identified. The control group exhibited higher level of hexanal compared to the other groups. Hexanal decreased in the presence of both SN and SCPN (Table 4). Hexanal is a marker of lipid oxidation and is formed from the oxidation of n-6 fatty acids such as arachidonic acid and linoleic acid [53]. On the other hand, the level of nonanal decreased in the presence of SCP (Table 4). This result is most likely due to certain antioxidant compounds present in chard powder [54,55,56].

In HTS samples, 3-hydroxy-2-butanone (acetoin) was identified, which is formed from the chemical oxidation of 2,3-butanediol produced during the metabolic activity of lactic acid bacteria [57]. In addition, acetoin can also be formed through pyruvate metabolism and from asparagine via oxaloacetate [58]. The highest value of 3-hydroxy-2-butanone was found in the control group. There was no statistically significant difference between the SN and SCPN groups (Table 4). This result indicates that nitrite inhibited the formation of this compound.

As shown in Table 3, the value of acetic acid decreased in the presence of nitrite (either synthetic or chard powder-derived), and no significant difference was determined between the groups in terms of this compound.

A total of 10 sulfur-containing compounds were identified in HTS samples. The lowest values of dimethyl disulfide and 3,3′-thiobis-1-propene were determined in the control group (Table 4). For allyl mercaptan, the SN group gave the highest value. This compound has also been reported in HTS [1,18,48,49]. While 2-propanethiol was not detected in the control group, it was identified in the nitrite-containing groups; however, no significant difference was observed between these groups regarding this compound (Table 4).

In the HTS groups, 26 terpene compounds were identified. Many studies have demonstrated that terpenes constitute the most important part, even more than 50%, of the volatile compounds determined in fermented dry sausages [1,48,51,59]. Among the terpene compounds identified in the present study, no statistical differences were found in the mean values of phellandral, camphor, o-cymene, camphene, β-pinene and α-thujene among all treatment groups. However, the use of nitrite source affected the other 20 terpene compounds, and except for β-phellandrene, and cumin aldehyde, the levels of all other compounds decreased with the use of chard powder. In addition, p-cymene, known as a peppery terpene, was not detected in the control group, and the use of chard powder also caused a reduction in this compound.

In the present study, only 2-pentylfuran, belonging to the furan group, was identified. This compound was not detected in the SN group, while the values determined in the control and SCPN groups did not show a statistically significant difference (Table 4). 2-pentylfuran is formed through lipid oxidation (linoleic acid and other ω-6 fatty acids) [60]. Moreover, due to its low sensory threshold and pleasant aroma, it contributes to flavor development in fermented sausages [61,62]. This compound has also been identified in HTS and other types of fermented sausages [1,48,49,51,63].

In the present study, four aromatic hydrocarbons were identified. SCPN and SN reduced the mean value of 1-methyl-1H-pyrrole. However, the difference between SN and SCPN was not statistically significant. 1-methyl-1H-pyrrole identified in this study has also been reported in other studies conducted on HTS [1,64].

**Figure 7:**
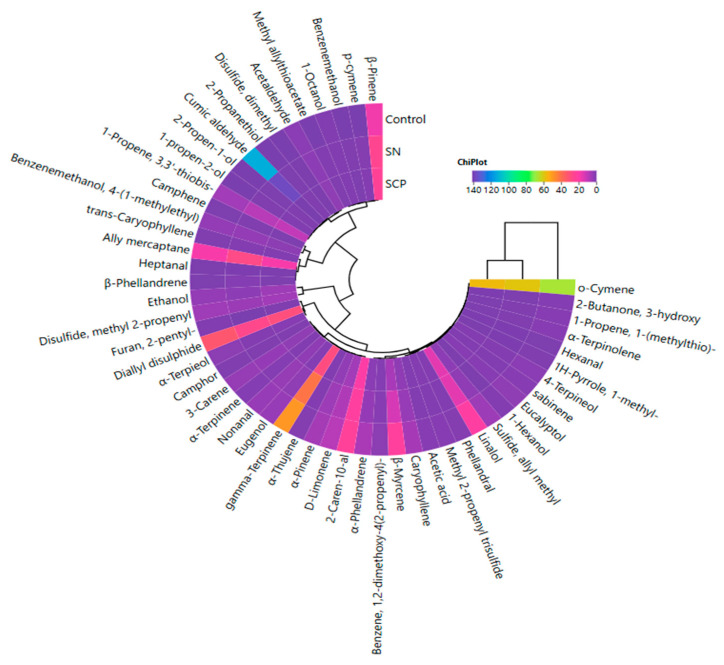
Cluster analysis of the correlation heat map illustrating the relationships between treatments and volatile compounds in HTS.

Figure 7 presents a correlation heat map illustrating the relationships between the groups and volatile compounds. The analysis revealed differences among the groups, with the use of different nitrite sources resulting in the formation of two distinct clusters. The control group was separated from the nitrite-treated groups, whereas the SN and SCPN groups clustered together, exhibiting closer correlations. Additionally, it was observed that the use of different nitrite sources did not cause substantial differences in volatile compound profiles (Figure 7).

**Figure 8:**
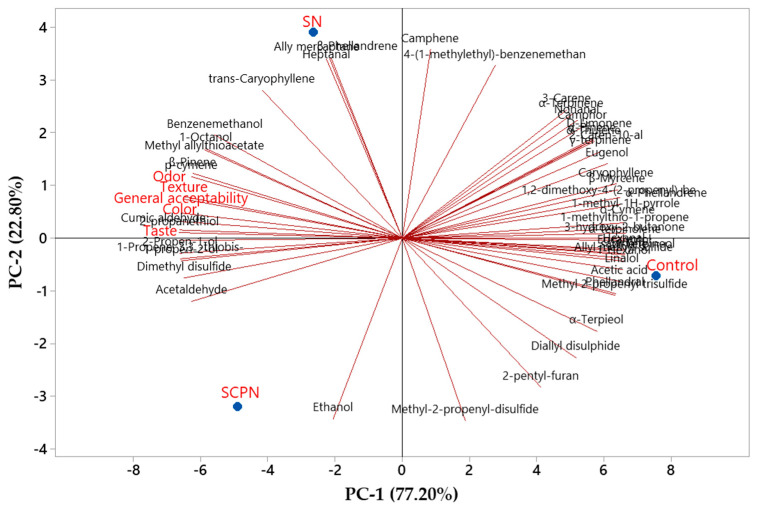
Principal component analysis biplot of the relationships between treatment, volatile compounds and sensory properties.

Principal component analysis (PCA) was conducted to assess the relationships among treatments, volatile compounds, and sensory attributes (Figure 8). The PC-1 explained 77.20% of the variation, and PC-2 explained 22.80% of the variation. The total variance was fully explained (100%) by the first two principal components. The SN and SCPN groups were on the negative side of PC-1 and showed a positive correlation. On the other hand, the control was on the positive side of PC-1 and showed a negative correlation with the SN and SCPN groups. The sensory parameters were located on the negative side of PC-1 and exhibited a positive correlation with the SN and SCPN groups. PCA results indicated that the SN and SCPN groups shared similar characteristics in terms of volatile compounds and sensory attributes.

## 4. Conclusions

The use of chard powder in HTS production generally did not have a negative effect on the overall characteristics of the products. The absence of nitrite resulted in significant decreases in the acceptability level of sensory attributes of the products. Furthermore, although many volatile compounds were affected by the use of SCPN, this effect was not at a level that would alter the sensory properties of the final product. However, to further clarify the effect of SCPN on the sensory attributes of HTS, studies on the use of different ingoing levels of SCPN are also important. On the other hand, in terms of residual nitrite, nitrite derived from SCP gave similar results to synthetic nitrite. Thus, it is thought that this study will contribute to clean label studies. However, although pH, a_w_, competition flora (starter cultures) and heat treatment are important hurdle effects in HTS, more studies are needed to investigate the effect of SCP on the behavior of foodborne pathogens. Future studies on the effect of this natural product on nitrosamines are also required.

## Figures and Tables

**Table 1:** Effect of using SCP on the physicochemical attributes of HTS.

Factors	pH	a_w_	TBARS (mg MDA/kg)	L*	a*	b*	Residual Nitrite (mg/kg) ^x^
Treatment (T)							
C	5.09 ± 0.39 ^b^	0.956 ± 0.015 ^a^	0.46 ± 0.14 ^a^	44.05 ± 4.65 ^b^	13.38 ± 2.79 ^b^	18.10 ± 1.81 ^a^	4.58 ± 1.01 ^b^
SN	5.10 ± 0.45 ^b^	0.957 ± 0.016 ^a^	0.38 ± 0.09 ^b^	45.43 ± 4.29 ^a^	15.02 ± 2.67 ^a^	17.47 ± 3.13 ^a^	12.78 ± 1.82 ^a^
SCPN	5.45 ± 0.25 ^a^	0.948 ± 0.020 ^b^	0.43 ± 0.12 ^a^	43.48 ± 3.05 ^b^	15.42 ± 2.62 ^a^	18.66 ± 3.33 ^a^	13.14 ± 2.36 ^a^
Significance	**	**	**	*	**	NS	**
Production Stages (PS)							
Batter	5.78 ± 0.07 ^a^	0.967 ± 0.004 ^a^	0.33 ± 0.03 ^c^	41.93 ± 2.15 ^c^	13.06 ± 2.75 ^c^	21.71 ± 1.88 ^a^	-
Fermentation	4.96 ± 0.20 ^d^	0.964 ± 0.002 ^b^	0.34 ± 0.04 ^c^	40.06 ± 2.21 ^d^	17.00 ± 1.68 ^a^	18.56 ± 1.92 ^b^	-
Heat Treatment	5.03 ± 0.22 ^c^	0.957 ± 0.003 ^c^	0.43 ± 0.07 ^b^	48.61 ± 2.05 ^a^	14.00 ± 2.99 ^b^	16.72 ± 0.85 ^c^	-
Drying	5.09 ± 0.23 ^b^	0.927 ± 0.008 ^d^	0.58 ± 0.08 ^a^	46.67 ± 1.84 ^b^	14.37 ± 2.13 ^b^	15.33 ± 0.80 ^d^	-
Significance	**	**	**	**	**	**	**-**
T × PS	**	**	*	NS	**	*	**-**

^a–d^: Different letters indicate statistical difference (*p* < 0.05) in each column. ** *p* < 0.01, * *p* < 0.05, NS: Not significant, C: Control, SN: 150 mg/kg Sodium nitrite, SCPN: Swiss chard powder as 150 mg/kg NaNO_2_ equivalent. ^x^: Nitrite analysis was performed only on the final product.

**Table 2:** Effect of using Swiss chard powder on the microbiological properties of HTS (log cfu/g).

Factors	Lactic Acid Bacteria	*Micrococcus/* *Staphylococcus*	*Enterobacteriaceae*	Yeast-Mold
Treatment (T)				
C	6.26 ± 2.01 ^a^	5.62 ± 1.47 ^a^	<2	<2
SN	6.12 ± 2.11 ^a^	5.78 ± 1.04 ^a^	<2	<2
SCPN	6.04 ± 2.11 ^a^	5.27 ± 1.51 ^b^	<2	<2
Significance	NS	*	NS	NS
Production Stages (PS)				
Batter	7.28 ± 0.15 ^b^	6.40 ± 0.14 ^b^	<2	<2
Fermentation	8.77 ± 0.07 ^a^	6.93 ± 0.12 ^a^	<2	<2
Heat Treatment	4.60 ± 0.34 ^c^	5.18 ± 0.27 ^c^	<2	<2
Drying	3.91 ± 0.46 ^d^	3.71 ± 0.88 ^d^	<2	<2
Significance	**	**	NS	NS
T × PS	*	NS	NS	NS

^a–d^: Different letters indicate statistical difference (*p* < 0.05) in each column. ** *p* < 0.01, * *p* < 0.05, NS: not significant, C: Control, SN: 150 mg/kg Sodium nitrite, SCPN: Swiss chard powder as 150 mg/kg NaNO_2_ equivalent.

**Table 3:** Effect of using SCP on the sensory attributes of HTS.

Factors	Odor	Color	Taste	Texture	General Acceptability
Treatment (T)					
C	5.61 ± 0.23 ^b^	4.59 ± 0.02 ^b^	5.44 ± 0.17 ^b^	5.59 ± 0.14 ^b^	5.48 ± 0.29 ^b^
SN	7.05 ± 0.17 ^a^	7.69 ± 0.24 ^a^	7.12 ± 0.12 ^a^	7.31 ± 0.36 ^a^	7.25 ± 0.30 ^a^
SCPN	6.97 ± 0.59 ^a^	8.00 ± 0.38 ^a^	7.52 ± 0.34 ^a^	7.24 ± 0.62 ^a^	7.33 ± 0.62 ^a^
Significance	*	**	**	**	*

^a,b^: Different letters indicate statistical difference (*p* < 0.05) in each column. ** *p* < 0.01. * *p* < 0.05. NS: Not significant. C: Control. SN: 150 mg/kg Sodium nitrite. SCPN: Swiss chard powder as 150 mg/kg NaNO_2_ equivalent.

**Table 4:** The effect of using SCP as a source of nitrite on volatile compounds of HTS (Au × 10^6^).

Volatile Compounds	RT	KI	RI	Treatment			
				C	SN	SCPN	Sign.
Alcohols							
Ethanol	8.680	539	A	3.44 ± 0.82 ^a^	2.59 ± 0.60 ^b^	4.86 ± 1.65 ^a^	**
1-propen-2-ol	9.927	543	B	0.00 ± 0.00 ^c^	0.59 ± 0.19 ^b^	0.83 ± 0.14 ^a^	**
2-propen-1-ol	10.401	581	B	0.00 ± 0.00 ^c^	0.46 ± 0.11 ^b^	0.62 ± 0.75 ^a^	**
1-hexanol	33.789	930	B	1.07 ± 0.21 ^a^	0.22 ± 0.05 ^b^	0.17 ± 0.03 ^b^	**
1-octanol	42.767	1127	B	0.80 ± 0.12 ^b^	1.04 ± 0.14 ^a^	0.96 ± 0.13 ^a^	*
Aldehydes							
Acetaldehyde	5.212	523	B	2.13 ± 1.08 ^a^	2.57 ± 0.87 ^a^	2.99 ± 0.91 ^a^	
Hexanal	28.762	850	A	0.44 ± 0.10 ^a^	0.32 ± 0.09 ^b^	0.30 ± 0.06 ^b^	*
Heptanal	34.982	955	A	0.18 ± 0.05 ^a^	0.31 ± 0.15 ^a^	0.17 ± 0.04 ^a^	NS
Nonanal	43.714	1143	A	4.11 ± 0.68 ^a^	3.82 ± 0.88 ^a^	2.76 ± 0.68 ^b^	**
Ketones							
3-hydroxy-2-butanone	24.728	779	A	1.31 ± 0.35 ^a^	0.48 ± 0.09 ^b^	0.30 ± 0.12 ^b^	**
Acids							
Acetic acid	19.032	710	A	1.51 ± 1.13 ^a^	1.00 ± 0.11 ^a^	1.03 ± 0.38 ^a^	NS
Sulfur Compounds							
2-propanethiol	10.401	570	B	0.00 ± 0.00 ^b^	0.47 ± 0.16 ^a^	0.56 ± 0.10 ^a^	**
Allyl mercaptan	13.970	574	B	18.70 ± 6.79 ^b^	28.05 ± 7.22 ^a^	18.39 ± 2.98 ^b^	*
Allyl methyl sulfide	20.655	730	B	4.79 ± 1.49 ^a^	2.79 ± 0.63 ^b^	2.64 ± 0.81 ^b^	**
1-methylthio-1-propene	23.315	732	C	1.52 ± 0.97 ^a^	0.68 ± 0.16 ^b^	0.43 ± 0.12 ^b^	**
Dimethyl disulfide	24.592	764	C	0.00 ± 0.00 ^b^	0.16 ± 0.06 ^a^	0.26 ± 0.12 ^a^	**
3,3′-thiobis-1-propene	31.789	888	B	5.35 ± 1.50 ^b^	8.66 ± 1.29 ^a^	9.98 ± 0.83 ^a^	**
Methyl-2-propenyl-disulfide	35.790	958	B	5.28 ± 1.02 ^a^	4.30 ± 0.94 ^a^	5.36 ± 1.09 ^a^	NS
Diallyl disulfide	43.169	1038	A	33.20 ± 7.01 ^a^	27.02 ± 4.14 ^a^	30.03 ± 5.35 ^a^	NS
Methyl allyl thioacetate	43.477	1045	C	0.00 ± 0.00 ^c^	0.96 ± 0.17 ^a^	0.66 ± 0.18 ^b^	**
Methyl-2-propenyl-trisulfide	45.164	1214	C	1.65 ± 0.83 ^a^	1.05 ± 0.17 ^a^	1.13 ± 0.23 ^a^	NS
Terpenes							
α-thujene	34.800	944	B	1.29 ± 0.38 ^a^	1.17 ± 0.16 ^a^	0.95 ± 0.26 ^a^	NS
α-pinene	35.384	950	B	5.63 ± 1.79 ^a^	4.83 ± 1.27 ^a^	3.18 ± 0.73 b	*
Camphene	36.521	970	B	0.43 ± 0.22 ^a^	0.54 ± 0.17 ^a^	0.34 ± 0.11 ^a^	NS
Sabinene	37.807	981	B	1.74 ± 0.74 ^a^	0.87 ± 0.08 ^b^	0.78 ± 0.18 ^b^	**
β-pinene	38.094	996	B	17.70 ± 6.45 ^a^	25.80 ± 5.48 ^a^	24.28 ± 3.54 ^a^	NS
β-myrcene	38.170	998	B	22.65 ± 5.38 ^a^	13.14 ± 4.35 ^b^	6.33 ± 0.69 ^c^	**
3-carene	39.270	1026	B	3.85 ± 0.75 ^a^	3.71 ± 0.43 ^ab^	2.88 ± 0.59 ^b^	*
α-phellandrene	39.456	1035	B	6.80 ± 1.96 ^a^	3.67 ± 1.66 ^b^	2.00 ± 1.03 ^b^	**
α-terpinene	39.845	1042	B	1.40 ± 0.43 ^a^	1.31 ± 0.49 ^ab^	0.91 ± 0.30 ^b^	*
D-limonene	40.310	1054	B	8.67 ± 2.83 ^a^	7.55 ± 1.86 ^ab^	5.31 ± 1.21 ^b^	*
o-cymene	40.492	1059	B	67.36 ± 9.92 ^a^	59.15 ± 20.82 ^a^	57.01 ± 10.05 ^a^	NS
β-phellandrene	40.614	1065	B	0.37 ± 0.19 ^b^	1.42 ± 1.42 ^a^	0.36 ± 0.08 ^b^	**
Eucalyptol	40.872	1075	B	2.92 ± 0.33 ^a^	1.69 ± 0.46 b	1.54 ± 0.28 ^b^	**
γ-terpinene	41.405	1103	B	49.20 ± 11.27 ^a^	41.38 ± 4.00 ^a^	29.43 ± 6.26 ^b^	**
α-terpinolene	42.492	1105	B	1.64 ± 0.67 ^a^	0.60 ± 0.12 ^b^	0.39 ± 0.08 ^b^	**
p-cymene	43.038	1147	B	0.00 ± 0.00 ^c^	1.29 ± 0.19 ^a^	1.07 ± 0.23 ^b^	**
Linalool	43.587	1161	B	21.01 ± 4.44 ^a^	15.24 ± 2.67 ^b^	15.21 ± 2.82 ^b^	**
Camphor	45.985	1230	B	0.69 ± 0.17 ^a^	0.66 ± 0.17 ^a^	0.55 ± 0.09 ^a^	NS
4-terpineol	46.171	1233	B	2.23 ± 0.67 ^a^	1.19 ± 0.28 ^b^	1.07 ± 0.26 ^b^	**
α-terpineol	46.712	1256	B	2.36 ± 0.43 ^a^	1.14 ± 0.15 ^b^	1.56 ± 0.42 ^b^	**
Cumin aldehyde	48.805	1334	B	112.98 ± 7.29 ^b^	137.72 ± 14.31 ^a^	141.67 ± 18.56 ^a^	**
Phellandral	50.023	1354	C	1.27 ± 0.29 ^a^	1.04 ± 0.62 ^a^	1.07 ± 0.34 ^a^	**
2-carene-10-al	50.450	1390	C	23.14 ± 1.70 ^a^	21.99 ± 2.29 ^ab^	19.69 ± 2.59 ^b^	NS
Eugenol	52.590	1456	B	4.18 ± 0.69 ^a^	2.88 ± 0.46 ^b^	1.33 ± 0.25 ^c^	**
Trans-caryophyllene	53.389	1473	B	0.68 ± 0.26 ^b^	1.14 ± 0.14 ^a^	0.83 ± 0.18 ^b^	**
Caryophyllene	54.244	1490	B	6.03 ± 0.54 ^a^	4.19 ± 0.43 ^b^	2.72 ± 0.29 ^c^	**
Furans							
2-pentylfuran	38.483	1021	B	0.84 ± 0.23 ^a^	0.00 ± 0.00 ^b^	0.57 ± 0.28 ^a^	**
Aromatic Hydrocarbons							
1-methyl-1H-pyrrole	25.023	786	B	0.66 ± 0.41 ^a^	0.29 ± 0.08 ^b^	0.16 ± 0.03 ^b^	**
Benzenemethanol	42.915	1132	B	0.00 ± 0.00 ^c^	0.64 ± 0.18 ^a^	0.39 ± 0.10 ^b^	**
4-(1-methylethyl)-benzenemethanol	50.230	1380	B	3.21 ± 1.32 ^a^	3.49 ± 0.76 ^a^	2.45 ± 0.26 ^a^	NS
1,2-dimethoxy-4-(2-propenyl)-benzene	53.182	1482	B	2.34 ± 0.72 ^a^	1.77 ± 0.59 ^ab^	1.42 ± 0.50 ^b^	*

^a–c^: Different letters indicate statistical difference (*p* < 0.05) in each column. * *p* < 0.05. ** *p* < 0.01. NS: Not significant. SD: standard deviation. Kovats index (KI) values were calculated for a DB-624 capillary column (J&W Scientific, 60 m × 0.25 mm i.d. × 1.4 μm film thickness) placed in a gas chromatograph equipped with a mass selective detector (MS). Reliability of identification (RI) was evaluated on a three-level scale according to the degree of confidence in compound identification: A: Mass spectrum and retention time are identical to those of an authentic reference standard; B: Mass spectrum and Kovats index are in agreement with those reported in the literature. C: Identification is based on the mass spectrum.

## Data Availability

The original contributions presented in this study are included in the article. Further inquiries can be directed to the corresponding author.
